# Mapping the genetic architecture of cortical morphology through neuroimaging: progress and perspectives

**DOI:** 10.1038/s41398-022-02193-5

**Published:** 2022-10-14

**Authors:** Dennis van der Meer, Tobias Kaufmann

**Affiliations:** 1 Norwegian Centre for Mental Disorders Research (NORMENT), Division of Mental Health and Addiction, Oslo University Hospital & Institute of Clinical Medicine, University of Oslo, Oslo, Norway; 2 School of Mental Health and Neuroscience, Faculty of Health, Medicine and Life Sciences, Maastricht University, Maastricht, The Netherlands; 3 Department of Psychiatry and Psychotherapy, Tübingen Center for Mental Health, University of Tübingen, Tübingen, Germany

**Keywords:** Genomics, Molecular neuroscience

## Abstract

Cortical morphology is a key determinant of cognitive ability and mental health. Its development is a highly intricate process spanning decades, involving the coordinated, localized expression of thousands of genes. We are now beginning to unravel the genetic architecture of cortical morphology, thanks to the recent availability of large-scale neuroimaging and genomic data and the development of powerful biostatistical tools. Here, we review the progress made in this field, providing an overview of the lessons learned from genetic studies of cortical volume, thickness, surface area, and folding as captured by neuroimaging. It is now clear that morphology is shaped by thousands of genetic variants, with effects that are region- and time-dependent, thereby challenging conventional study approaches. The most recent genome-wide association studies have started discovering common genetic variants influencing cortical thickness and surface area, yet together these explain only a fraction of the high heritability of these measures. Further, the impact of rare variants and non-additive effects remains elusive. There are indications that the quickly increasing availability of data from whole-genome sequencing and large, deeply phenotyped population cohorts across the lifespan will enable us to uncover much of the missing heritability in the upcoming years. Novel approaches leveraging shared information across measures will accelerate this process by providing substantial increases in statistical power, together with more accurate mapping of genetic relationships. Important challenges remain, including better representation of understudied demographic groups, integration of other ‘omics data, and mapping of effects from gene to brain to behavior across the lifespan.

## Introduction

The cerebral cortex, the outermost sheet of neurons of the cerebrum, is responsible for processing and integration of multimodal sensory information, higher-order cognitive functions such as planning, and the execution of behavioral strategies through the initiation of movement [1, 2]. As such, its functioning determines much of an individual’s ability to reach life goals, with its development having been named ‘the crowning achievement of evolution’ [3]. Any structural abnormalities may convey problems with sensory processing, impaired cognitive abilities, and maladaptive behavior, covering many of the symptoms associated with brain disorders. Indeed, all prevalent brain disorders have been linked to deviant cortical morphology [4–8]. A more complete picture of the determinants of cortical morphology is therefore essential to prevent or treat these disorders as well as gain a better understanding of human cognition and behavior in general.

The size and shape of the cortex changes significantly throughout the lifespan [9], determined by tightly regulated patterns of expression of thousands of genes, varying over regions and over time [10]. Neuroimaging, in particular magnetic resonance imaging (MRI), allows for a range of objective and highly reproducible measures to be derived non-invasively from an individual, allowing us to study cortical morphology in vivo. In addition, enormous advances in genomics technology [11], combined with large-scale biobanking efforts and a push towards open science [12], have led to the availability of data that allows us to start unravelling its complex genetic architecture, i.e., the characteristics of the genetic variation responsible for its heritable phenotypic variability [13]. Here, we review the progress made in this field of research, with a particular emphasis on findings from genetic studies of cortical morphology measured through MRI that inform us on these characteristics. After a brief introduction on the neurobiology of the cortex, we summarize the current state of the art together with important theoretical and methodological considerations, and end with thoughts on future research directions.

## Cortical anatomy in a nutshell

The cerebral cortex is a thin sheet of grey matter, containing neuronal bodies, along the surface of the brain, making up 42% of the total brain mass [14]. Cortical neurons, a mixture of long-range glutamatergic projection neurons and short-range GABAergic interneurons, are first generated from various subtypes of progenitor cells in transient embryonic zones near the surface of the lateral ventricles [15]. As these differentiate and migrate to their final destination in the cortex, radially and tangentially, they become organized into ontogenetic columns perpendicular to its surface [16]. According to the well-established radial unit model, the number of columns determines surface area, while the amount of cells within a column determines the thickness of the sheet [16]. The large surface area of humans compared to other species, without a concomitant increase in thickness, is thereby explained by mutations that lead to a larger pool of neuronal stem cells, causing an exponential increase in columns [3, 14]. Regionalization, organizing the cortex into histologically distinct areas, takes place during embryonic and early life stages [16]. This is accomplished through gradients in the expression of transcription factors under the influence of morphogens released by patterning centres organized along three principal axes, bringing about areal fate by regulating the expression of cell-surface molecules and synaptic organization [17]. The vast majority of the developing cortex will become isocortex, also called neocortex [18], consisting of six horizontal layers, known as laminae, while the remainder becomes allocortex, located mediorostral, with three or four laminae. Through mechanical forces, the cortex folds into highly consistent patterns of gyri and sulci [19], allowing for greater surface area to fit in the cranial vault while reducing distance between neurons, enhancing signal transmission [20].

As a result of its intricate organizational processes, there are widespread differences in cell type, number, and density across the cortex, reflecting functional specializations [21]. Capturing the corresponding regional differences in genetic architecture would therefore bring about valuable information about their different contributions to perception, cognition, and behaviour. This requires us to differentiate between areas by devising borders based on some micro- or macroscale organizational feature. See Box 1 for an overview of commonly used parcellation strategies for MRI-based studies.

Box 1 Parcellating the cortex Cortical morphology is most commonly studied through T_1_-weighted MRI scans, with research scanners typically providing a resolution of 1 mm^3^. Pre-processing of these images is most commonly achieved through highly automated and standardized pipelines provided by popular software suites such as FreeSurfer [140], optimizing reproducibility and comparability between studies. These pipelines capture inter-individual morphological differences by registering images onto another, enabling group comparisons. Surpassing the initial voxel-based morphometry approaches, current surface-based pre-processing approaches allow for the cortical sheet to be reliably extracted and represented by hundreds of thousands of data points known as vertices.

While vertex-wise studies take optimal advantage of the amount of information provided by these images, most studies employ some form of data reduction to lower computational burden or for ease of interpretation, aggregating over vertices within a certain predefined area. Ideally, the borders of these areas are drawn in a way that maximizes differences in some biological characteristic of interest, thereby optimizing signal and interpretability. The enormous complexity of the cortex makes this no easy task, with organizational properties stretching across multiple levels and along multiple axes. Choice of feature, from micro- (e.g., cytoarchitecture) to macrostructural (e.g., functional connectivity), and algorithm (boundary-based versus clustering), as well as granularity (number of parcels) will each have substantial impact on the delineation, and thereby on outcome of studies employing a certain parcellation [141].

Study comparability and standardization promote the use of common parcellations that are not necessarily optimal for individual study purposes. Currently, the Desikan-Killiany atlas is the most common cortical parcellation strategy. It divides the cortex into 34 regions per hemisphere, based on gyral and sulcal patterns [133]. A notable alternative is the Human Connectome Project atlas from Glasser et al. consisting of 360 parcels, delineated by local changes in structure and activity, derived from multiple data modalities [142].

Combining vertices with similar genetic architectures can capture the strong role of genetics in regional differentiation, optimizing parcellations for genetic studies. Using twin data, Chen et al. applied fuzzy cluster analysis to pairwise genetic correlations across the cortex to make area- and thickness-specific atlases [96, 97]. While corroborating some lobar and regional borders from non-genetic parcellations, this approach also suggested divisions clearly deviating from those based on structure and function, and revealed a strong hierarchical and modular genetic organization. Further, while area primarily showed differences along an anterior-posterior axis with greatest similarity between clusters in the same lobe, thickness differences were along a dorsal-ventral axis with closest relations between clusters with similar maturational timing [96, 97].

## Genetic architecture of cortical morphology

The genetic architecture of a trait describes the characteristics of the genetic variation that explain its broad sense heritability, encompassing additive effects of common variants (primarily single nucleotide polymorphisms; SNPs), rare variants, and non-additive effects including gene-environment interactions [13]. Characteristics such as the number of genetic determinants, known as polygenicity, and the effect sizes involved, also referred to as a trait’s discoverability [22], are essential knowledge for genetic studies as they dictate the required design and analysis approaches.

**Fig. 1:**
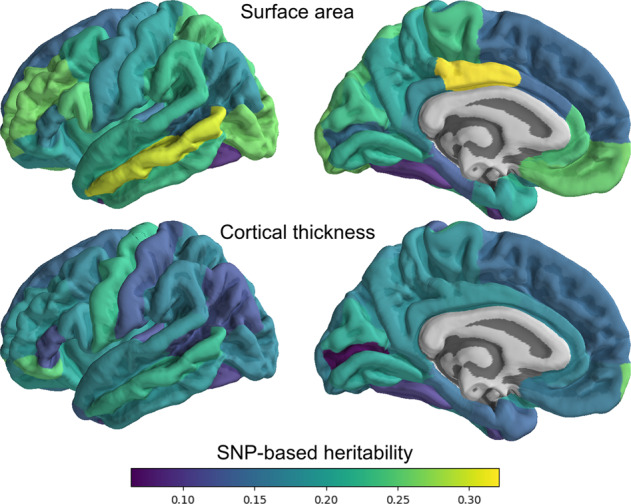
Regional SNP-based heritability across the cortex, with the lateral (left) and medial (right) views for surface area at the top and cortical thickness at the bottom row. The estimates are calculated through LD score regression [132], applied to MRI data from 33,735 White European individuals as described previously [115], and parcellated according to the widely-used Desikan Killiany atlas [133]. As indicated through the colour-coding, heritability ranges from below 0.10 to above 0.30, with the regional estimates for surface area showing a wider spread than cortical thickness.

A range of twin and family studies have proven conclusively that all common metrics of cortical morphology are highly heritable [23, 24]. Close to half of this heritability is due to additive effects of common variants [25], similar to other complex traits. The most general metric is cortical grey matter volume, between the pia mater and the white matter, totalling approximately 0.35–0.5 litre in the average human brain [26]. This volume is determined by the amount and size of neurons, dendrites, and glial cells that make up the sheet. Pedigree studies have indicated that cortical volume has a broad heritability of 0.7, with regional measures ranging from 0.2 to 0.8 [24, 26], while its estimated SNP-based heritability is 0.30 [27]. Common estimation approaches for cortical volume have some notable issues [28]. Further, volume is the product of the thickness of the sheet and its surface area, suggesting that it is more informative to study these two constituent metrics instead [29]. Cortical thickness, on average about 2.6 mm across the brain [16], has a broad heritability of 0.8 [30], and a SNP-based heritability of about 0.26 [25]. The broad heritability of surface area, in total about 0.15 cubic metre [26], is estimated at 0.9 [30], with common variants explaining 0.34 of the phenotypic variance [25]. Regional measures of heritability for either of these metrics tend to be somewhat lower, as shown in Fig. 1. Measures of cortical morphology that capture folding patterns are less commonly investigated, despite associations with brain disorders and cognitive performance beyond those captured by area and thickness [31–33]. The gyrification index, the ratio of total area to exposed area (‘convex hull’), in humans averaging about 2.5 [19], has an estimated broad heritability of 0.7 [34]. Sulcal depth is an inherently vertex-level measure, reflecting the convexity of any given point on the surface [35]. Its broad heritability varies widely across the cortex, ranging from 0 in higher-order regions to 0.8 along the major sulci [36].

**Fig. 2:**
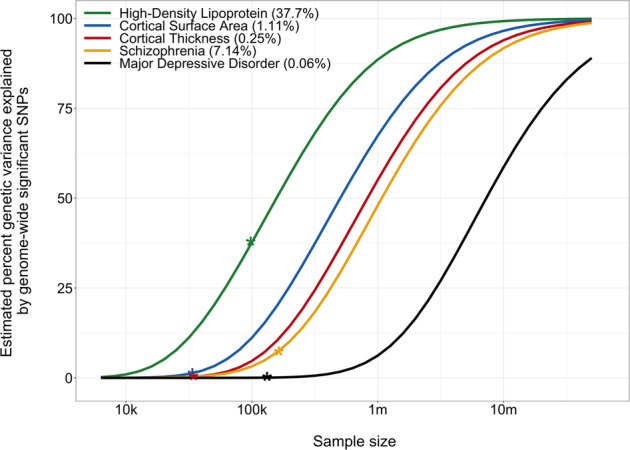
Relation between sample size and proportion variance explained by genome-wide significant variants. This iss calculated through MiXeR [93] applied to summary statistics of recently conducted GWAS, with sample sizes indicated by stars [134–137].

A major motivation for imaging genomics is that brain measures are thought to capture processes mostly intermediate on the causal path from genetics to brain disorders, i.e., more proximal to genetic effects [37]. It is also plausible that the genetic determinants of objective and reliable MRI-derived measures are easier to find than ill-defined diagnoses based on subjective measures. However, it is now clear that the genetic architecture of cortical morphology is nearly as complex as that of brain disorders, with high polygenicity and low discoverability [38]. These two fundamental characteristics, the product of which corresponds to heritability, can be quantified based on the distribution of genetic associations observed through GWAS, e.g., by applying Gaussian mixture modelling to the summary statistics [22]. Using this technique, we have estimated that area and thickness both involve several thousand causal variants, with each individual variant on average explaining a small fraction of one percent of the heritability [38]. This is about one order of magnitude lower polygenicity than prevalent brain disorders, as also confirmed through other approaches [39, 40], yet higher than e.g., biochemical measures such as high-density lipoprotein [22]. Regional area measures are on average more heritable and discoverable than regional thickness, which fits with the fact that larger number of genetic variants have been discovered for area than for thickness [25]. There is also more variation in discoverability between regions for area, i.e., its architecture appears more region-specific than that of thickness [38], in line with the notion that cortical columns are the functional units of the cortex [3]. The polygenicity and discoverability of a trait determine the required GWAS sample sizes to explain a given proportion of genetic variance by whole-genome significant variants. From the data shown in Fig. 2, we can extrapolate that explaining half of the heritability of surface area will require half a million subjects, i.e., current imaging genetics studies are still grossly underpowered, as also described in Box 2. Well-defined measures that capture the effects of a limited set of biological processes should have relatively low polygenicity and high discoverability, and therefore require smaller sample sizes. As such, these estimates may guide selection of metrics and parcellation schemes with greater specificity, boosting the ability of genetics studies to find the biological pathways involved. Corroborating these statements is the fact that regional area estimates produced through the genetically-informed Chen et al. parcellation had significant higher discoverability than estimates following other parcellations [38].

Box 2 Overcoming low statistical power through collaboration The small genetic effect sizes involved in complex traits, including cortical morphology, necessitate large sample sizes for detection [143]. Exacerbated by the multiple comparison correction for running a million independent tests [144], GWAS requires tens of thousands of observations to identify even the most impactful common variants (none of which explain individually more than 0.5% variance in brain morphology) [145], and hundreds of thousands to capture a substantial proportion of the total genetic variance, as evident from Fig. 2. Analogously, finding reproducible associations between brain and behavior in MRI studies, at high spatial resolution, has been shown to require thousands of scans [146].

MRI data is relatively costly to obtain, with few individual labs able to collect more than a few hundred scans. For this reason, researchers have started forming consortia, pooling their data. Most notable in this context is the Enhancing NeuroImaging Genetics through Meta-Analysis (ENIGMA) consortium, formed in 2009, bringing together an ever-increasing group of researchers from hundreds of institutions across the world. ENIGMA has aggregated tens of thousands of scans, using meta-analysis and harmonized processing protocols to overcome the large between-site variability while allowing primary data analyses to remain on-site to satisfy data protection regulations [145]. As such, this consortium has generated the first successful, large-scale GWAS of cortical thickness and area [25].

Available sample sizes have increased enormously recently due to large-scale biobanking efforts, combined with a push for open science. The UK Biobank is the prime example of this, having genotyped and deeply phenotyped half a million British volunteers, accessible for researchers all over the world [147, 148]. Multimodal MRI data collection is underway; at the time of writing, half of the targeted sample size of a hundred thousand individuals has been scanned [149]. The Adolescent Brain Cognitive Development (ABCD) study is another valuable resource, creating an American population-representative cohort of over ten thousands genotyped children aged 9–10 at recruitment, undergoing MRI scans every two year [150]. Initiatives such as the UKB and ABCD, therefore, promise unprecedented insights into the development of the cortex and its genetic underpinnings.

## Finding the genetic determinants

Genetic architecture metrics, such as heritability, describe overall characteristics of a trait, while discovery of the specific variants involved provide more mechanistic insights. Evolutionary genomics analyses have been instrumental in identifying the most central genes and molecular pathways underlying cortical development [3, 15]. An overview of the many components of these pathways is beyond the scope of this review, and they have been summarized elsewhere [41]. Generally, animal models allowed for the identification of mammalian orthologs of invertebrate development such as the homeobox and hedgehog genes, found to regulate fundamental cortical developmental processes [42]. In humans, early linkage studies of severe malformations of cortical development, e.g., lissencephaly, further implicated dozens of genes regulating neuronal proliferation, migration and cortical organization [43]. The first imaging genetic studies at the beginning of this century investigated polymorphisms in candidate genes, particularly those encoding neurotransmitter receptors or transporters [44], e.g., *COMT*, 5*-HTT* or *DAT1*, or known disease genes such as *Apo-E4* [45] and *DISC1* [46], finding that these polymorphisms influence cortical thickness and area in the general population.

Since its introduction nearly two decades ago, GWAS has become the predominant approach to investigate complex traits genetics [47, 48]. Contrary to candidate gene studies, GWAS is an explorative mass-univariate approach, testing the individual, additive effect of millions of common variants across the genome on the outcome of interest. While this necessitates a harsh multiple comparison correction that limits statistical power, it better serves the ultimate goal of comprehensive genetic mapping, given the involvement of thousands of variants. GWAS output by itself carries little actionable information, yet the summary statistics it produces enable numerous valuable post-GWAS analyses [49]; the distribution of effect sizes informs us about the genetic architecture of a trait, functional annotation points to biological pathways involved, and comparisons with other traits can indicate the strength and nature of their genetic relationships.

Only recently have enough MRI scans become available to allow for large-scale GWAS of cortical morphology [25, 27, 40, 50]. Following early GWAS of single cortical measures in small samples [51, 52], the ENIGMA consortium (see Box 2) has published the largest such GWAS to date, including global and regional measures from over fifty thousand individuals [25]. Analysis of total area plus 34 regional measures led to the discovery of 187 unique loci, while mean and regional thickness measures produced a total of 50 loci. Most of the 12 loci identified for total area had been previously linked to intracranial volume and brain disorders. Mapped genes and pathway analyses further implicated primarily early neurodevelopmental processes. The two loci associated with mean thickness were similarly coupled to processes such as neurogenesis and neuronal migration. Most of the loci found to influence the regional measures, correcting for the global measures, were associated with only a single region, i.e., the findings corroborated the presence of regional specificity in genetic determinants. Many regional loci were mapped to genes with known roles in brain development, most prominently the Wnt signalling cascade, which is central to progenitor cell proliferation and areal identity [53]. Parallel to the ENIGMA study, the CHARGE consortium conducted a GWAS on the same set of global and regional measures, as well as cortical volume, with a discovery sample size of 23 thousand individuals [27]. They reported the same general patterns, with more loci discovered for area than for thickness. The findings for area and volume mostly overlapped, and implicated genes involved in neurodevelopment. Also notable is the GWAS statistics released by the UK Biobank core neuroimaging team for nearly four thousand different brain measures, across structural, functional and white matter modalities [54]. While this broad approach limited the depth of investigation and increased the multiple comparison burden, it did provide a wealth of information, including insight into how much signal each of these measures produce, relative to each other. For instance, the results indicated that the relatively understudied cortical grey-white matter contrast may be a particularly interesting metric to study in greater detail, given it had the highest heritability and highest locus yield. Last, we have recently conducted a GWAS on twelve regional measures of area and thickness, following the Chen et al. parcellation strategy. Combining the adult UKB and adolescent ABCD cohorts totalling nearly fifty thousand individuals, we found 467 loci surviving multiple comparisons, achieving a substantially higher yield than studies using other parcellations, demonstrating this parcellation’s greater discoverability [38]. Generalization of findings within the UKB cohort to ABCD was high, supporting the notion of strong genetic control over cortical regionalization early in life [40]. This is further in line with the pathways found to be significant, similar to those found through the ENIGMA, relating to early neurodevelopmental processes.

While ever-increasing sample sizes will move us along the discovery curve shown in Fig. 2, openly available resources, databases, and approaches for functional annotation truly advance our understanding of the biological pathways shaping cortical morphology. As the common variants included in GWAS tag numerous other variants through complex patterns of linkage disequilibrium, sophisticated fine mapping approaches have enabled identification of variants within a genomic region most likely to underlie functional consequences [55]. Gene mapping has improved by integrating knowledge on associations with expression levels and chromatin interactions [56]. Coupling of mapped genes to previous GWAS findings, as well as tissue-specific expression data and tests for overrepresentation among a wide array of gene sets have firmly established that morphology measures are primarily shaped by early neurodevelopmental processes, as well as revealed the strength of involvement of specific molecular pathways such as Wnt signalling [25]. Implication of biologically informative genetic pathways thereby requires less power than discovery of individual variants [57], due to the aggregation of effects and lower multiple comparison burden [58].

Beyond GWAS, rare genetic variants have been identified with sizeable effects on cortical morphology. For complex traits, there is an overall negative relation between frequency and effect size of variants [59]. In that regard, copy number variants (CNVs), i.e., deletions or duplications of segments of the genome that often span several genes [60], tend to have substantially larger effects on morphology than any common variant [61]. Consequently, whereas the most impactful common variants increase odds of a brain disorder by no more than 25%, CNVs may convey over a tenfold higher risk [61]. They are known to be important driving forces in evolution, and appear especially relevant for explaining the rapid expansion of the human cortex; compared to other primates, humans have obtained multiple copies of genes that regulate cortical neurogenesis and radial migration [62]. Further, CNVs are like natural genetic experiments, deleting or duplicating genes, providing insights into the function of these genes and eluding to mechanistic relationships between traits, given carriers often suffer from a constellation of impairments. Interestingly, with some CNVs, both deletion and duplications are risk factors for the same disorder, while copy number effects on brain measures follow a dose response [61]. For instance, we have shown that 15q11.2 deletion carriers have thicker cortices and smaller surface area, while duplication carriers showed the opposite pattern, mediating the association of this CNV with cognition [63]. It is further clear that CNVs are especially risk factors for neurodevelopmental disorders [64, 65], once again emphasizing the particularly strong role of genetics in early cortical development.

Beside CNVs, rare variants may be investigated through whole-exome (WES) or whole-genome sequencing (WGS). This is important as the high penetrance of rare variants may explain much individual variation, and a subset of common genetic variant signals may result from synthetic associations with these variants [66]. WES restricts sequencing to the 1% of the genome that contains coding regions, thought to contain the majority of disease-causing variants, making it substantially cheaper and manageable than WGS data. However, the benefits of better coverage by WGS are beginning to outweigh the continuously dropping costs for sequencing and computation [67], with its use further stimulated by numerous findings suggesting substantial regulatory roles for non-coding regions [68]. Promisingly, in a pilot study, Wainschtein et al. reported that nearly all of the heritability of height and body-mass index could be recovered from WGS data, with the majority being explained by variants with a minor allele frequency between 0.0001 and 0.1 [69]. This type of data is now rapidly becoming available, including for the half million UKB participants [70, 71], suggesting that a WGS study in the near future could provide a treasure trove of new information on the genetic architecture of cortical morphology.

Non-additive genetic effects on cortical morphology, making up the final component of broad heritability, have been scarcely investigated. There have been findings of epistasis [72], i.e., a genetic variant moderating the effects of other variants [73], although their validity has been questioned, due to concerns about inflated test statistics [74]. Age is a highly likely candidate to interact with genetic variation, given the trajectories of cortical measures over the lifespan [9], together with substantial changes in gene expression [10]. The rate of change of brain measures, including global cortical area and thickness, derived from longitudinal MRI scans has been shown to be heritable, with a GWAS on fifteen thousand individuals discovering several variants with robust age-dependent effects [75]. Sex is another important demographic with far-reaching impact on cortical morphology, and the genetic underpinnings of brain disorders have been shown to differ, somewhat, between men and women [76]. While there is substantial evidence for widespread sex effects on cortical gene expression [77], there are no major reports of gene-by-sex interactions on morphology. Additionally, the central role of environmental adaptation in evolution strongly suggests that genetic variants regulate our response to a wide array of stimuli, yet there is little overall evidence that gene-environment interactions play a significant role in explaining the heritability of complex traits, with only scattered reports of interactions involving candidate genes impacting grey matter volume [78]. The lack of robust findings may be in part explained by the fact that the detection of statistical interactions is highly dependent on model and measurement characteristics, making them notoriously difficult to reliably detect and interpret [79], exacerbating the low statistical power of current genetic studies. Of interest in this context is a little-known strategy applied by our group to identify variance-controlling loci; despite a limited GWAS sample size we successfully discovered variants impacting phenotypic variability of cortical thickness, not associated with mean thickness [80]. Ensemble-learning approaches such as random forest regression, incorporating all higher-order interactions between predictors, may further provide fruitful ways to explore the extent of non-additive effects [81, 82].

## Gene expression mediates genetic effects on cortical morphology across the lifespan

**Fig. 3:**
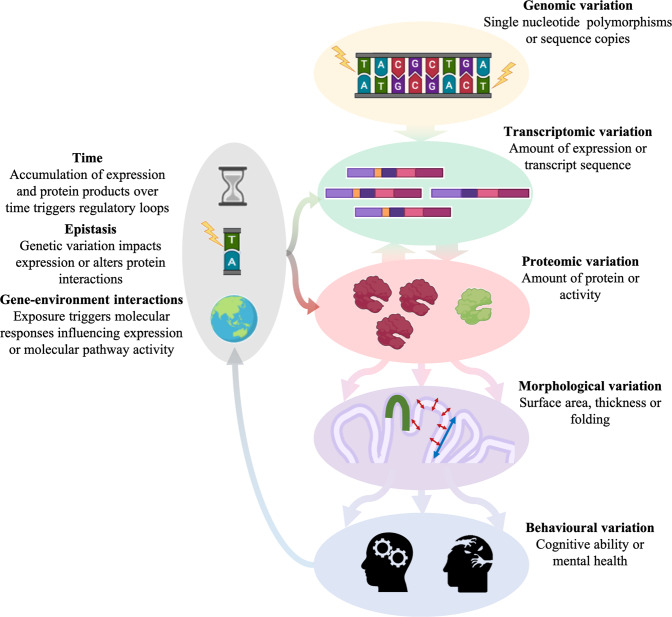
Visualization of the pathway from gene to brain to behaviour, reflecting how variation at each level influences the next. Biological complexity increases as we move further down, away from the direct effects of genetic variation, given the accumulation of influences. The wide range of measures we use to study complex traits, be it cortical morphology or behavior, can be further split into more fine-grained measures capturing additional levels of organization, e.g., total surface area into regional measures, or diagnoses into symptoms. The complex relations and hierarchical structure ensure there is extensive pleiotropy between such traits, horizontally and vertically [138].

Genetic variation forms the basis for interindividual differences in cortical morphology; causal variants influence either the expression levels of genes or alter characteristics of their protein products. Either way, the activity of molecular pathways is altered, changing cellular functioning, which in turn influences neuronal circuitry. The ultimate impact of genetic variants is therefore mediated by multiple interacting layers of regulation, varying across regions and time, further moderated by environmental influences, see Fig. 3. This makes clear that knowledge of functional genomics, charting the regulation and coordination of genes that determine the activity of biological processes, is needed to gain a more complete understanding of cortical development.

Maturation of the cortex during neurodevelopment, generating its intricate circuitry and regional functional specialization, results from highly regulated patterns of gene expression [10]. Genetic variation has been proven to contribute to the variability of expression in the cortex [83, 84]. Approximately 82% of genes are expressed in the cortex, of which 85% are differentially expressed across time and/or regions [10], testifying to the importance of adopting a lifespan perspective and local tissue samplings.

The difference in gene expression patterns between pre- and postnatal cortical tissue is large [10, 85]. Prenatal changes in cortical expression levels over time are an order of magnitude larger than postnatally, and between-subject correlations are also much higher early in life [85], indicating that the tight regulation of fundamental neurodevelopmental processes gives way for individualization later in life [86]. The expression of genes involved in cell division decreases from conception onwards, those related to synaptic functioning increase throughout infancy, while genes regulating axonal functioning increase prenatally and then decrease after birth [85]. This matches well-known temporal patterns of major neuron differentiation and pruning processes [87], attesting to the fact that data on gene expression can be queried to map intricate neurobiological mechanisms over time.

Expression across cortical regions is relatively homogeneous, compared to the amount of differential expression over time or between subcortical regions [10, 88]. Based on their expression profiles, regions within the same lobe cluster together, i.e., there is a smooth spatial gradient of expression across the cortex, with the highly specialized primary visual cortex being most distinctive [10, 88]. Correlations between regions further increase with age [10], reflecting that transcriptional differences are most pronounced when functional specialization is formed during early development. Subsets of spatially co-expressed genes can further be coupled to specific cell types, such as neurons, oligodendrocytes, astrocytes and microglia [89].

One of the key findings from the ENIGMA GWAS of cortical morphology was that the common variation associated with either total area or mean thickness was related to developmental phase-specific gene regulation that contrasted the two measures [25]. Both showed enriched heritability in genomic regions regulating brain tissue, yet area showed enrichment primarily within progenitor cell types and mid-fetal–specific active regulatory elements while thickness was enriched in adult-specific elements. This reiterates the necessity for a lifespan perspective in genetic studies as well, as the effects of genetic variation will depend on when the genes are expressed.

Taken together, these findings indicate that gene regulatory patterns are highly age- and region-specific, arising from complex combinations of factors over time that cannot be fully explained by simple additive effects as identified through GWAS on gross morphological features. Studies of single-cell or nucleus RNA-seq data can provide additional, more nuanced insights, by mapping cell type-specific signalling and gene expression patterns that explain important developmental processes [90]. Further, the use of cortical organoids, modelling development across the lifespan, allows for valuable transcriptomic and epigenomic data to further enhance our understanding of these processes [91].

## Genetic relationships between metrics and regions

Charting the shared and specific genetic influences on different metrics of morphology, rather than studying them in isolation, brings us a more complete understanding of how the cortex produces human behavior. It is however crucial to consider the assumptions and limitations of the statistical techniques employed. The currently predominant approaches are geared towards emphasizing the differences between measures, neglecting the extent of shared information in these measures that may be leveraged to improve our ability to uncover their genetic architectures.

**Fig. 4:**
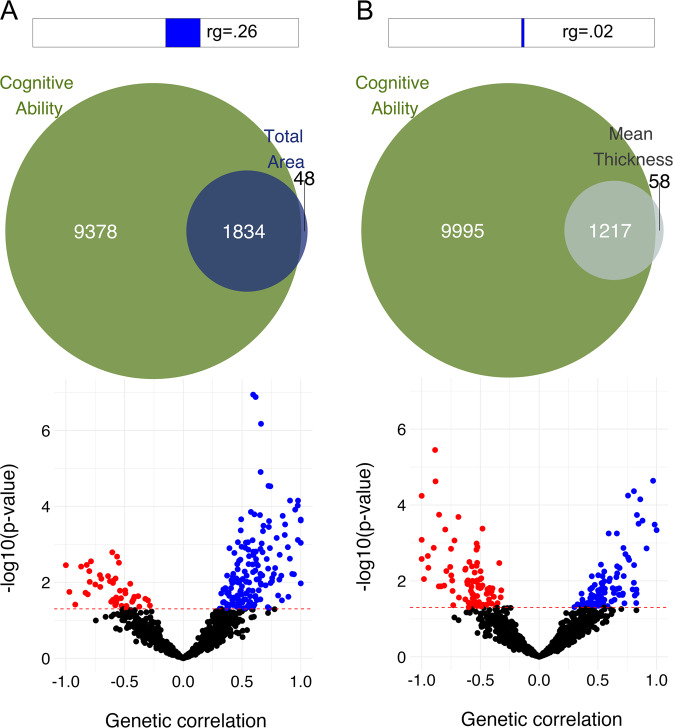
Genetic overlap of cortical measures with cognitive ability, measured through complementary approaches. At the top are the low estimates of global genetic correlation, calculated through LD score regression [92], between GWAS of cognitive ability [139] and total surface area (**A**) and mean thickness (**B**) [115]. The Venn diagrams below it reflect the amount of causal variants estimated to contribute to each of the traits as well as their overlap, regardless of effect directions, calculated through bivariate Gaussian mixture modelling [93]. At the bottom, volcano plots showing the results from Local Analysis of [co]Variant Annotation (LAVA) [94]. This explains the discrepancy between the two global metrics of overlap, as a mixture of opposing directions of local correlations will cancel each other out at the global level.

Surface area and thickness have been widely proclaimed to be genetically independent of each other and therefore recommended to be studied separately [26, 30]. These claims are based on a robust negligible genetic correlation as calculated through tools such as linkage-disequilibrium score regression [92]. While these metrics are indeed likely to have a substantial non-overlapping genetic component, as supported by the radial unit model, characterizing them as predominantly independent may be misguided. The issue here lies in the reliance on a global estimate of correlation as an indicator of independence, summarizing the overall coherence of the strength and direction of effects across millions of genetic variants. Given high polygenicity, it is nearly certain that there is a mixture of effect directions, with some variants having a significant positive or negative effect on both metrics while others lead to lower thickness and larger area or vice versa. This is confirmed by approaches estimating genetic correlations between complex traits for small chunks of DNA across the genome, consistently producing a large number of both positive and negative local correlations, even for pairs of complex traits assumed to be independent [93, 94]. When aggregating, effects with mixed directions will cancel each other out, ensuring global correlations will virtually always underestimate the true amount of genetic overlap. By using Gaussian mixture modelling to estimate the number of overlapping causal variants regardless of directions of effects, we have shown that the majority of variants influencing area and thickness are overlapping [38]. It should be noted though that the higher degree of overlap that can be detected by these and similar methods comes at the cost of lower information about the nature of the relationship between the traits studied, precisely because they are agnostic about the directions of effects. These estimates may therefore best be complemented with other metrics of (local and global) overlap, each providing additional information (see Fig. 4).

Similarly, regional measures must share to a large extent the same biology, being made up of the same overall cell types and with the same gross laminar organization, reflecting they are constituents of one overarching system. It is however common practice to statistically correct for a ‘global’, e.g., total area when studying regional area, thereby emphasizing regional differences. Genetic correlations tend to closely match phenotypic correlations, with neighbouring regions generally having positive correlations while those further away are negatively correlated, if corrected for the global measure. When uncorrected, these estimates shift to being all positive [95]. The strength of correlations thereby follow an anterior-posterior gradient for area, and a dorsal-ventral gradient for thickness, as reflected in the Chen et al. parcellations [96, 97].

Mixed effect directions and statistical corrections for global measures thus obscure the large extent of shared biology between different metrics and regional measures, with substantial implications for study design. The major GWA studies of cortical morphology have investigated each measure individually [25, 27, 50], necessitating a multiple comparison correction. This may be complemented by a multivariate approach, leveraging the shared information between all measures that together capture cortical morphology. At the cost of lower interpretability and loss of information about directions of effects, such an approach can boost discovery of genetic variants threefold when jointly analyzing the same sets of regional measures, identifying hundreds of loci with half the sample size [98]. Discovery goes up tenfold when aggregating even more information by studying vertex-wise data, uncovering thousands of loci [99, 100], that also replicate better out-of-sample than when employing univariate approaches [101]. A computationally efficient multivariate approach further facilitates the genetic study of vertex-wise cortical measures not well-captured by parcellation into ROIs, such as sulcal depth. Indeed, this understudied metric of cortical folding, linked to several brain disorders and cognition [31, 102, 103], was found to be both more heritable and discoverable than area and thickness [100], suggesting it is a relatively untapped resource for understanding cortical morphology. Each of these multivariate GWAS studies thereby found 5–10% of all genes to be significantly associated with cortical morphology, while explaining 10–30% of genetic variance (cf. Fig. 2), suggesting that with large enough sample sizes GWAS findings will largely corroborate the omnigenic model of complex traits, positing that all tissue-expressed genes contribute to a certain extent [104]. As such, aided by the power of multivariate approaches, focus will shift from the dichotomy of discovery towards charting the strength of contributions and how this varies across component measures, creating a more nuanced, comprehensive map of the genetic architecture of cortical morphology.

## Genetic relationships with clinical and cognitive traits

One of the ultimate goals of imaging genomics is to uncover the neurobiological pathways underlying brain disorders, to identify biomarkers and facilitate the development of strategies to prevent or treat disorders by manipulating these pathways [105, 106]. Generally, highly heritable psychiatric disorders [107] such as schizophrenia, bipolar disorder, and major depression are associated with smaller area and thinner cortices [4–6]. Cognitive ability is associated with larger area and, somewhat less consistently, thicker cortex, with regional patterns dependent on the specific task and age of the participants [108, 109]. Trajectories of morphological changes over time may thereby be better predictors than absolute size [110], in line with the often-reported finding that the heritability of cognitive performance increases with age [111]. Despite their high heritability and strong associations with cortical morphology, studies have reported little to no genetic correlation of these traits with brain measures [112], with the largest point estimate, between educational attainment and surface area, being 0.2 [25]. As with the relation between surface area and thickness, the true extent of genetic overlap will be underestimated due to mixed directions of effects of genetic variants. (see Fig. 4). Estimates ignoring effect directions indicate nearly all genetic variants underlying surface area and thickness are involved in schizophrenia [113]. This genetic overlap may also be leveraged to enhance the discovery of genetic variants; [114] e.g., conditioning schizophrenia GWAS summary statistics on brain morphology GWAS data through a Bayesian framework enabled us to identify twice as many loci as the original schizophrenia GWAS, with the mapped genes being highly differentially expressed in brain tissue [115]. Ideally, the pathway from gene to brain to behaviour is charted, e.g., through mediation analyses [78], and tools now exist that can carry out such analyses at the large-scale GWAS level [116]. Mendelian randomization analyses have complemented the mediation framework by providing concrete evidence that cortical morphology indeed is on the causal path to brain disorders [40]. The possibility of reverse causation should be kept in mind though, whereby disease processes or environmental influences following a diagnosis explain a portion of observed cortical differences [117], the extent of which remains unclear.

## Going beyond associations: individualized prediction

Another major goal of human genetics, distinct from attaining mechanistic insights, is to be able to accurately predict traits. Polygenic scores approximate an individual´s genetic propensity for a trait by aggregating the additive effect sizes of all significant common genetic variants as determined through GWAS, multiplied by the individual’s allele count [118, 119]. These scores as of yet do not allow for clinically relevant prediction of complex traits, often explaining single digit percentages in phenotypic variance [120], reflecting the fact that the current generation of GWAS is underpowered [121], producing noisy effect size estimates for genetically heterogeneous traits. Given their complex relationship, with mixed directions of effects and negligible genetic correlations, polygenic scores of cortical morphology are even more unlikely to predict disorders and vice versa. However, these scores may inform the genetic relationships between brain and behaviour [122], distinguish biological subgroups, or serve as a tool for risk stratification [119]. Further, prioritizing variants based on the strength of evidence for shared effects on both the disorder and cortical morphology may improve the performance of these scores [115]. This prioritization is likely to weed out some false positives as it requires evidence for the involvement of a variant in two separate studies, plus it may create scores that tag a more specific set of biological processes, those determining overlap between traits.

Fundamentally, polygenic score performance depends on the accuracy of the estimated effect sizes. While the increasingly larger sample sizes will help with this, accuracy is ultimately determined by the homogeneity and similarity of the composition of the training sample with the test sample; differences in factors that influence the relation between genetic variation and the trait, such as ethnic background, will therefore lower performance [120, 123]. The underrepresentation of non-White Europeans has been receiving increased attention in psychiatric genomics in recent years [124], but less so in imaging genetics. Performance may further be boosted by including additional sources of information when calculating these scores, such as comorbidities as well as non-additive effects, e.g., with age. In that regard, normative modelling approaches are notable, providing individual-level statistics on deviations from a normative age-trajectory for a population of interest [125]. Incorporating the influence of rare variants is also likely to provide a substantial improvement, as these are particularly relevant for the individual [126]. Last, alternative prediction approaches better suited for complex, noisy data than standard regression will be valuable as they continue to be developed, improving their current low reproducibility. This includes ensemble-learning approaches and deep neural networks applications [127].

## Conclusions

Our knowledge of the genetic architecture of cortical morphology has increased enormously in the last decade, spurred on by ever-increasing sample sizes and the development of more appropriate, powerful biostatistical approaches to match its complex characteristics. It is now clear that all measures of cortical morphology that can be accurately derived from MRI scans are highly heritable and polygenic with low discoverability, making them valuable but challenging intermediate phenotypes for brain disorders.

Studies investigating its genetic determinants have only scratched the cortical surface so far, with the ‘missing heritability’ still being substantially larger than the amount of explained heritability. There is good progress in identifying additive effects of common genetic variants, yet this is the most easily discoverable tip of the iceberg. Together with better-powered GWAS, the quickly upcoming availability of WGS data should enable us to map the majority of variance explained by additive effects on common MRI-based metrics of morphology before the end of this decade, as has been achieved recently for other complex traits [57]. A greater challenge lies in the detection of non-additive effects, both between variants as well as effects over the lifespan and interactions with demographics and environmental factors. Interaction effects require substantially more statistical power to detect, while data on most moderators will only be available in a subset of current cohorts [79, 128]. Perhaps most pressing here is mapping effects through longitudinal data to capture cortical development. The near future looks promising in this regard as well though; large ongoing initiatives such as ABCD and UKB are collecting and releasing longitudinal MRI data at a rapid pace, and powerful tools for large-scale brain-wide and genome-wide analyses of data with dependency structures have become available recently [116].

Future studies would do well to include multiple metrics to gain a more complete picture of the genetic architecture of cortical morphology, through appropriate analytical approaches. The vast majority of studies only focus on area and/or thickness, neglecting informative metrics such as folding and grey-white matter contrast. Besides providing complementary information and insight into genetic relationships, joint analyses benefit from greater statistical power due to the large degree of shared information missed by estimates of global genetic correlations [114]. As illustrated by the findings from a series of recent studies from multiple research groups showcasing a new generation of biostatistical tools, leveraging genetic overlap has the potential to substantially enhance both locus discovery and genetics-based prediction [98, 129, 130].

Important challenges consist of combined analyses of metrics, at a higher spatial resolution and over the lifespan. More knowledge on the impact of demographics, particularly ethnic background, is needed to ensure findings ultimately better benefit those most in need [120, 123]. Last, uncovering genetic determinants only solves one piece of the puzzle; A true understanding of cortical development and its role in human behavior will require a multi-omics approach, integrating genomics with epigenomics, transcriptomics and proteomics to discover how these regulate molecular, cellular, and neural processes over time [131], fully characterizing the path from gene to brain to behavior.
